# Annotations

**Published:** 1910-06-11

**Authors:** 


### ANNOTATIONS.

### Two Veterans.

On the first day of June the medical profession lost two very old and eminent members by the deaths of Sir Francis Seymour Haden and Dr. Elizabeth Blackwell. Sir Francis Haden, who had reached the advanced age of ninety-two, was a Fellow of the Royal College of Surgeons, and his chief professional activities were as surgeon in the Guards, and subsequently as a very highly successful practitioner in Belgravia; his professional education was received at University College, London. He had been honorary surgeon to the Department of Science and Art, South Kensington, but attained even greater fame as an artist than in his own profession; etching was the branch of art in which he stood pre-eminent. Sir Francis Haden was Founder and First President of the Royal Society of Painter-Etchers, and was the author of several works on art; he was also an honorary member of the Société des Artistes Français, of the Institut de France, and of the Academie des Beaux Arts. The deceased surgeon became a K.C.B. in 1894. Miss Blackwell had attained very nearly as many years as Sir Francis Haden. Born in England, she accompanied her parents to America in her youth and began to study medicine at Ashville, U.S.A., in 1845. After being refused entrance to some of the American medical schools she gained admission to the Geneva University in New York State, and graduated in 1849. In 1851 she founded a hospital and school of medicine for women; later on she came to England, made the acquaintance of Miss Nightingale, and was placed upon the Register in 1859 after studying at St. Bartholomew's Hospital. Later on she took part in the foundation of the London School of Medicine for Women, and was the founder of the National Health Society of London. Her admission to the profession in England preceded by eleven years that of the two ladies next in seniority to Miss Blackwell—namely, Miss Jex-Blake and Mrs. Garrett Anderson.

### The Provision of Meals Act.

The report on the working of the Education (Provision of Meals) Act, 1906, up to March 31, 1909, has been recently issued by the Board of Education. We gather from its pages that the total number of meals supplied elsewhere than in London was 2,751,326 in 1907-8 and 9,671,789 in 1908-9. In arriving at the average number of meals supplied to each child fed, the reports of those authorities are disregarded who have not stated the number of children fed. It is then found that outside London the average number of meals supplied to each child fed amounted in 1907-8 to 41 and in 1908-9 to 61.6. The total cost in 1908-9 in England and Wales, exclusive of London, was £69,527, of which £17,393 was provided by voluntary contributions, £295 contributed by or recovered from parents, administrative expenses, including official salaries, amounting to £3,559. In the return made by the London County Council it appears that the total expenditure for London under the Act during the same period amounted to £20,972, of which £438 was received from voluntary contributions, £40 was contributed by or recovered from parents, the expenses of administration, including official salaries, amounting to £1,250. The weekly average of children fed by this authority was 39,632, for whom 4,546,771 meals were provided at an average cost of 2d. per meal. Mr. Selby-Bigge, who provides a prefatory note to the report, is of opinion that one of the most useful by-products of this Act, and of the medical provisions of the Act of 1907, is the stimulus given to the formation of local "children's care committees" and similar voluntary agencies, which, by the help of the children under their care, are able to obtain influence in the home and thus to combat ignorance and prejudice. Among those teachers who have superintended the distribution, the opinion is general that much improvement in manners and cleanliness of the children is to be noticed as the result of meals taken in common with the cheerful surroundings of clean linen, flowers, and plants.

### Prevention of Abortion in France.

M. Louis Barthon, Minister of Justice, proposes to modify Article 317 of the Penal code which deals with the prevention of abortion. He is of opinion that legislation actually in force is no longer capable of coping with the trend of modern ideas, and that repressive measures become more and more uncertain in their effects as the law now stands. Thus in 1905, out of fifty-four accused only three were imprisoned, and thirty-eight were acquitted. In 1906 and 1907 the proportion of acquitted to accused stood at much the same figure, and in 1908 only one out of sixty-six accused was sent to prison. The Minister asks the sanction of Parliament to impose a penalty of from six months' to three years' imprisonment, with a fine of from 100 to 3,000 francs, on anyone who, by administering foodstuffs, potions, or medicines, or by violence or any other means whatsoever, has procured abortion in any pregnant woman, such penalties to apply equally to any woman who has procured abortion by any means upon herself. He proposes also to increase the penalties in the case of doctors, surgeons, health officers, and pharmacists who have indicated or made use of the means of procuring abortion. Lastly, he seeks power under the proposed law to inflict the above-mentioned penalties of fine and imprisonment on any person who at a public meeting or in a public place shall have indicated the means of procuring abortion, or on any person who shall have caused the dissemination of manuscript, printed matter, books, advertisements, designs, engravings, pictures, remedies, instruments, or any other objects relative to abortion, whether these have been sold or exposed for sale, even in private, or exposed, distributed, or advertised either in public or through the post, or by domiciliary visitation, or any other method of transport, the penalties to be inflicted whether the advice, etc., so given was effective or otherwise.

